# Haplotype analysis of key genes governing grain yield and quality traits across 3K RG panel reveals scope for the development of tailor‐made rice with enhanced genetic gains

**DOI:** 10.1111/pbi.13087

**Published:** 2019-02-15

**Authors:** Ragavendran Abbai, Vikas Kumar Singh, Vishnu Varthini Nachimuthu, Pallavi Sinha, Ramchander Selvaraj, Abhilash Kumar Vipparla, Arun Kumar Singh, Uma Maheshwar Singh, Rajeev K. Varshney, Arvind Kumar

**Affiliations:** ^1^ International Rice Research Institute South‐Asia Hub, ICRISAT Campus Hyderabad India; ^2^ Centre of Excellence in Genomics and Systems Biology International Crops Research Institute for the Semi‐Arid Tropics Hyderabad India; ^3^ International Rice Research Institute Metro Manila Philippines

**Keywords:** 3K RG panel, cloned genes, haplotype mining, *haplotype‐based breeding*

## Abstract

Though several genes governing various major traits have been reported in rice, their superior haplotype combinations for developing ideal variety remains elusive. In this study, haplotype analysis of 120 previously functionally characterized genes, influencing grain yield (87 genes) and grain quality (33 genes) revealed significant variations in the 3K rice genome (RG) panel. For selected genes, meta‐expression analysis using already available datasets along with co‐expression network provided insights at systems level. Also, we conducted candidate gene based association study for the 120 genes and identified 21 strongly associated genes governing 10‐grain yield and quality traits. We report superior haplotypes upon phenotyping the subset of 3K RG panel, *SD1*‐H8 with haplotype frequency (HF) of 30.13% in 3K RG panel, *MOC1*‐H9 (HF: 23.08%), *IPA1*‐H14 (HF: 6.64%), *DEP3*‐H2 (HF: 5.59%), *DEP1*‐H2 (HF: 37.53%), *SP1*‐H3 (HF: 5.05%), *LAX1*‐H5 (HF: 1.56%), *LP*‐H13 (3.64%), *OSH1*‐H4 (5.52%), *PHD1*‐H14 (HF: 15.21%), *AGO7*‐H15 (HF: 3.33%), *ROC5*‐H2 (31.42%), *RSR1*‐H8 (HF: 4.20%) and *OsNAS3*‐H2 (HF: 1.00%). For heading date, *Ghd7*‐H8 (HF: 3.08%), *TOB1*‐H10 (HF: 4.60%) flowered early, *Ghd7*‐H14 (HF: 42.60%), *TRX1*‐H9 (HF: 27.97%), *OsVIL3*‐H14 (HF: 1.72%) for medium duration flowering, while *Ghd7*‐H6 (HF: 1.65%), *SNB*‐H9 (HF: 9.35%) were late flowering. *GS5*‐H4 (HF: 65.84%) attributed slender, *GS5*‐H5 (HF: 29.00%), *GW2*‐H2 (HF: 4.13%) were medium slender and *GS5*‐H9 (HF: 2.15%) for bold grains. Furthermore, haplotype analysis explained possible genetic basis for superiority of selected mega‐varieties. Overall, this study suggests the possibility for developing next‐generation tailor‐made rice with superior haplotype combinations of target genes suiting future food and nutritional demands via *haplotype‐based breeding*.

## Introduction

Domestication of plants marks one of the most ancient events in the human civilization. Since then, efforts are continuously being made at various levels to ensure that every stomach is fed with quality food, worldwide. Rice is the staple food for more than half of the population, and in the context of the rapidly increasing population, there is an obvious need to enhance grain yield under fluctuating climatic conditions. The advances in genomics and other allied fields render a great opportunity for translational research in plants (Varshney *et al*., 2014). Several key genes associated with rice grain yield and related traits have been functionally characterized in the past. Various high‐throughput OMICs platforms have been established in the past which aided in the functional characterization of several key genes controlling major traits in rice (Li *et al*., 2018). About 2296 genes associated with various traits such as rice grain yield (189 genes), growth and development (513 genes), disease resistance (221 genes), nutrient‐use efficiency (207 genes), fertility (174 genes), floral organ and heading date (276 genes), phytohormone (472 genes), insect resistance (31 genes), grain quality (63 genes), stress responsiveness (367 genes) etc., are cloned and functionally validated (Wing *et al*., 2018). For instance, *OsSPL14* perturbs miR156‐mediated regulation leading to a rice plant with higher yield and increased lodging resistance (Jiao *et al*., 2010). *Gn1a*, is a gene for cytokinin oxidase/dehydrogenase (*OsCKX2*), an enzyme that degrades the phytohormone, cytokinin (Ashikari *et al*., 2005). Reduced expression of *OsCKX2* leads to cytokinin accumulation in inflorescence meristems increases the number of reproductive organs, which ultimately enhances grain yield. Crowell *et al*. (2016) quantified 49 panicle phenotypes in 242 tropical rice accessions with the imaging platform PANorama and identified 10 candidate genes in pathways known to regulate plant architecture. Recently, it has been demonstrated that through marker‐assisted selection (MAS) *OsSPL14* WFP allele dramatically increased grain yield/panicle by 10.6–59.3% in four different genetic backgrounds (Kim *et al*., 2018). Similarly, genome editing approach was used to alter two yield attributing genes namely, *Gn1a* and *DEP1*. Analysis of genome edited lines revealed one mutant allele of *Gn1a* and three of *DEP1* confers superior yield than other natural high‐yield alleles (Huang *et al*., 2018).

The generated genomic resources, especially the re‐sequencing of various diverse germplasm lines, would aid in identifying and exploring allelic/haplotype variations, thus harnessing genetic diversity. Ultimately, this would pave the way for the identification of novel donors and novel alleles associated with the traits of interest, which can in turn be deployed in crop improvement (Varshney *et al*., 2018). The rice gene bank collections serve as a potential source of allelic diversity for important genes. For example, haplotype diversity of *GW2* locus was investigated in 93 *indica* and aromatic rice germplasm and based on the re‐sequencing data of the *GW2* locus four new SNPs were identified (Dixit *et al*., 2013). Recently, Singh *et al*. (2017) investigated the SNP haplotype variation in *GS3* gene for grain size (major grain size gene) in a set of 160 wild rice accessions that resulted in three major (*GS3*‐H1, *GS3*‐H4, and *GS3*‐H9) and six minor (*GS3*‐H2, *GS3*‐H3, *GS3*‐H5, *GS3*‐H6, *GS3*‐H7, and *GS3*‐H8) haplotypes.

In this context, the 3K RG re‐sequencing project holds great promise for harnessing genetic diversity in rice (Li *et al*., 2014). The identified superior versions of genes could be combined via recently established, fast and robust ‘haplotype assembly’ concept (Bevan *et al*., 2017). Although many genes governing major traits have been already identified, still the information regarding the superior haplotype combinations of various key genes in rice is lacking. In this study, we analysed the haplotype diversity of about 120 previously characterized major genes that influence grain yield and grain quality in rice across the entire 3K RG panel. Also, the performance of various haplotypes of 21 associated genes governing 10 major grain yield and quality related traits was evaluated across two seasons in the subset of 3K RG panel. Furthermore, we identified superior haplotypes for each documented trait which could be employed in *haplotype‐based breeding*. Taken together, we report the possibility of developing ‘tailored rice’ suiting future needs by introgression/assembly of superior haplotypes into any genetic background.

## Results

### Haplotype analysis of 120 cloned key genes confirms rich genetic diversity across 3K RG panel

**Figure 1:**
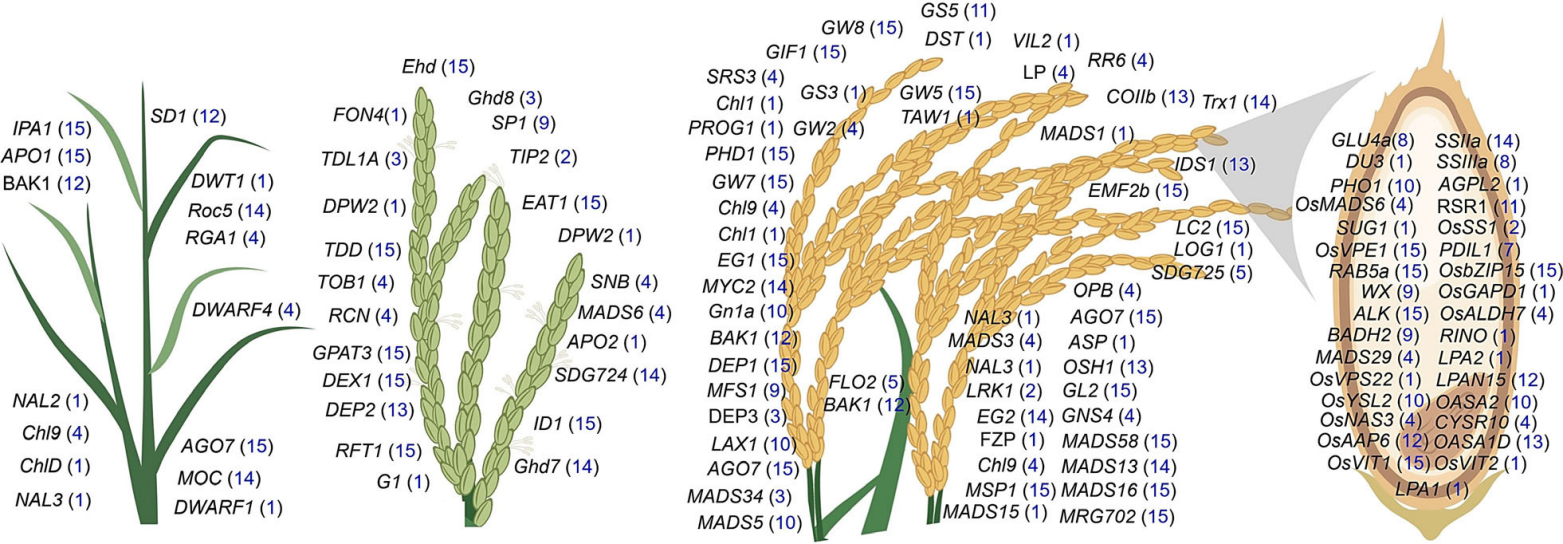
Haplotype analysis of 120 cloned key genes associated with major grain yield and quality related traits in rice. The chosen 120 genes were previously functionally characterized and were reported to govern/regulate several major grain yield (87 genes) and quality related traits (33 genes) such as tiller number, flowering, panicle architecture, lodging resistance, single plant yield, grain amylose content, grain Fe and Zn concentration, grain size etc. For all these genes haplotype analysis was conducted and the numbers in blue within brackets indicates the number of haplotypes for that particular gene in the 3K RG panel. Interestingly, more than 75% of the genes had haplotypes ranging from 2 to 15 among the 3024 lines. This is a gold mine for the identification of superior haplotypes and utilizing the same in developing elite versions of rice via *haplotype‐based breeding*.

The 3K RG panel is a gold mine that would enable the designing of ‘Tailored Rice’ suiting future's food and nutritional demands via *haplotype‐based breeding*. In this study, haplotype analysis has been conducted for a total of 120 previously functionally characterized key genes controlling grain yield (87 genes) and grain quality (33 genes) traits across the entire 3K RG panel (Table S1). Interestingly, about 92 of the 120 analysed genes had haplotypes ranging from 2 to 15, while the remaining 28 genes had only one haplotype in the 3K RG panel (Figure 1). For instance, *OsSPL14* had about 15 haplotypes, *OsSS1* was with two haplo‐groups, while *OsMADS1* had only one haplotype across the 3K RG panel. Candidate gene based association study of 120 genes revealed that a total 21 of these previously cloned genes were strongly associated with the target grain yield and quality traits (Table S2). The effect of identified haplotypes (Table S3) for these 21 genes affecting 10 major traits such as plant height (*SD1*), number of tillers (*MOC1* & *IPA1*), days to flowering (*Ghd7, SNB, OsVIL3, TOB1* & *TRX1*), panicle length (*DEP3, DEP1* & *SP1*), primary branch numbers per panicle (*LAX1, LP* & *OSH1*), grain yield (*PHD1, AGO7* & *ROC5*), grain size (*GS5* & *GW2*), grain amylose content (*RSR1*), grain Fe and Zn (*OsNAS3*) concentration were validated in the subset of the 3K RG panel across two crop seasons (WS2017 & DS2018) and the study revealed superior performance of some of the haplotypes of the studied genes over others.

### Haplotype frequency of target genes

The haplotype frequency of *SD1*‐H7, *SD1*‐H10, *SD1*‐H11 and *SD1*‐H12 was as low as 0.03%, while *SD1*‐H9 was the highest with 57.14%. HF of *MOC1*‐H8 was only 0.03%, whereas *MOC1*‐H4 was present across 52.18% of the lines. Similarly, *Ghd7*‐H13 had 0.33% and *Ghd7*‐H14 was 42.56% regarding haplotype frequency. In the same way, *DEP3*‐H3 (HF: 1.42%), *LAX1*‐H6 (HF: 0.03%), *PHD1*‐H3 (0.03%), *GS5*‐H6 (HF: 0.03%), *RSR1*‐H5 (0.26%) and *OsNAS3*‐H2 (1.00%) had the minimum frequency for the respective genes. On the other hand, maximum haplotype frequency was recorded for *DEP3*‐H1 (HF: 92.99%), *LAX1*‐H8 (64.28%), *PHD1*‐H2 (72.19%), *GS5*‐H4 (HF: 65.84%), *RSR1*‐H1 (58.76%) and *OsNAS3*‐H1 (96.3%) for the particular genes (Figure S1a‐i). In addition, the haplotype frequency of *AGO7‐*H15 was 3.33%, *IPA1*‐H14 was 6.64%, *DEP1*‐H2 was about 37.53%, *LP*‐H13 was 3.64%, *SNB*‐H9 was 9.35%, *GW2*‐H2 was 4.13% etc., (Table S3).

### Meta‐expression analysis provides insights into the spatiotemporal expression profile and co‐expression network of selected target genes

As a first step towards understanding the relationship between some of the previously characterized key genes associated with the target traits, their expression profile *viz.,* spatiotemporal profile and expression during various developmental stages (Figure S2) were analysed using publically available datasets encompassing transcriptomes of over 1800 rice samples. As a result, we found that *SD1* expressed strongly in stem elongation and heading stage. *Ghd7* a major gene for heading date had the highest expression in booting stage and was predominantly expressed in flag leaves. Also, strong expression of *DEP3* was witnessed in the panicle and peduncle and similarly *LAX1* was also highly expressed in the panicle. It was found that *PHD1* expressed strongly in leaves, flag leaves and panicle branch. *GS5* expressed the most in booting stage and panicle branch. The highest expression of *RSR1* was found in the flag leaf and at heading stage, while *OsNAS3* expressed predominantly in panicle branch and during milk stage of grain filling.

Furthermore, co‐expression network for the selected genes provided insights into the functional nature of positively co‐expressed genes. A stringent criterion (PCC > 0.9) was used for determining the co‐expressed genes and network construction to increase the reliability. In the case of *Ghd7*, the top 10 positively co‐expressed genes were related to regulation, protein degradation and stress‐responsiveness (Figure S3a,b). The co‐expressed genes of *OsNAS3* were associated with regulation of transcription, enzymatic activities and protein degradation (Figure S3c,d). In both cases, some of the genes were unclassified. Similar kind of analysis has been performed for other target genes as well (Figure S4a–f, Table S4). Genes associated with reproduction co‐expressed commonly with *SD1*, *DEP3* and *LAX1*. Most of the genes co‐expressed with *PHD1* were related to metabolic processes of plastid. Regarding co‐expression network of *GS5*, genes regulating enzymatic activity such as kinase activity, hydrolase activity was present. Finally, genes related to carbohydrate metabolic processes were one of the co‐expressed genes with *RSR1*.

### Identification of superior haplotypes for *haplotype‐based breeding*

#### Development of subset panel of 3K genome

**Figure 2:**
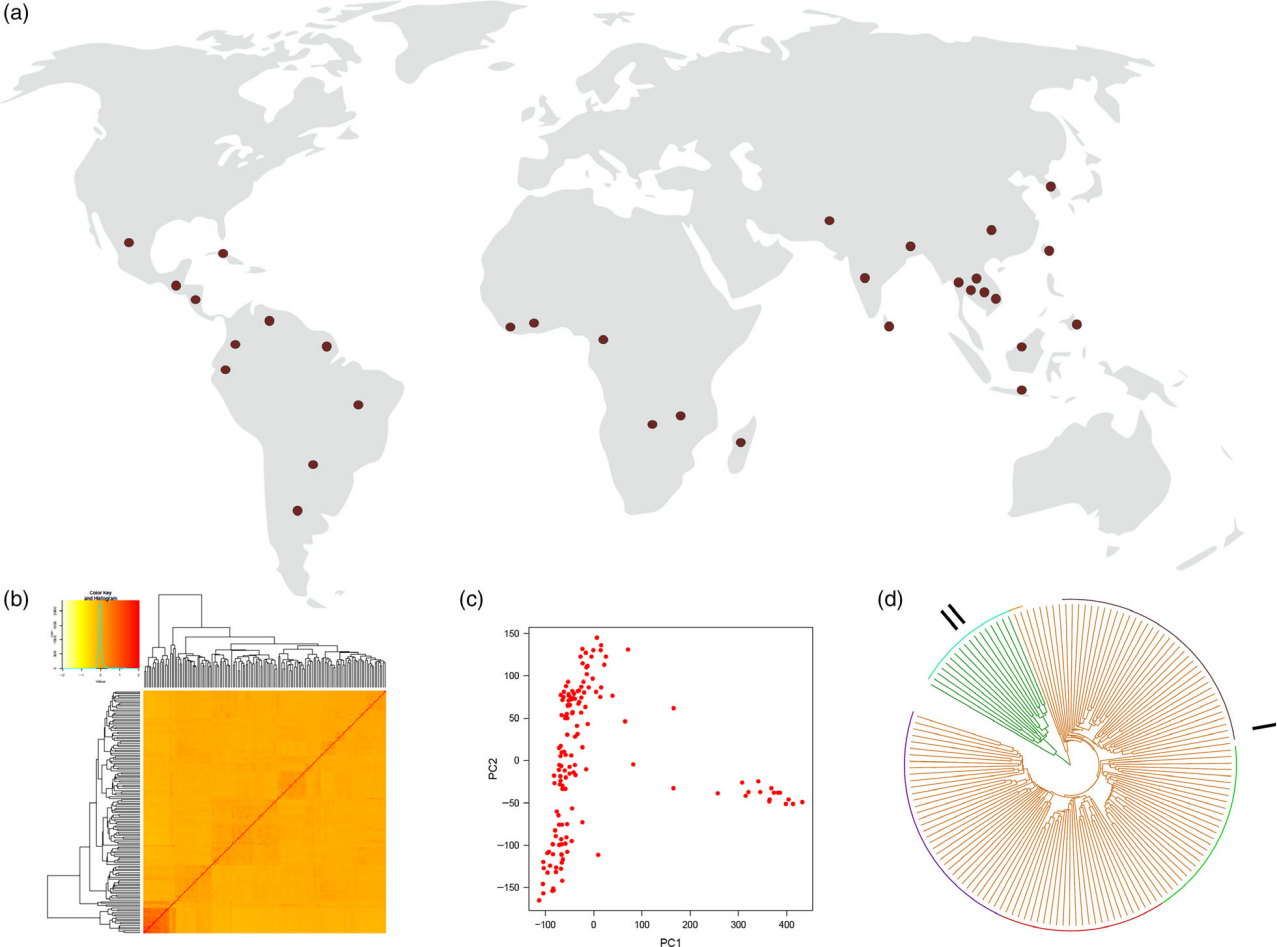
Establishing subset of 3K RG panel. A core panel comprising of 150 lines was developed based on diversity analysis using 559, 297 SNPs. (a) The chosen lines were from diverse geographical locations (32 countries). (b) Kinship among the 150 lines. (c) Two significant principle components were found in the panel. (d) SNP based UPGMA tree reveals the presence of two major clusters with five subgroups within the first major cluster and two subgroups in second major cluster.

A subset of 3K RG panel was developed in such a way that most of the diversity of the entire panel is captured. The subset consists of 150 lines originating from 32 countries (Figure 2a) and the SNP based diversity analysis provided insights into the sequence‐based diversity of the panel (Figure 2b,c). The diversity analysis was carried out using genome‐wide 559K SNP data points and unweighted pair group method with arithmetic mean (UPGMA) tree was constructed using the calculated dissimilarity index (Figure 2d). Popular hierarchical clustering method that is unweighted pair group method with arithmetic mean (UPGMA) was used to determine genetic distance from molecular data. Unweighted mean of the distances between all pairs of accessions was calculated and clusters are formed based on its nearness. Dendrogram obtained from UPGMA has resulted in two major clusters. Based on the grouping of accessions, a set of 150 diverse lines was chosen as the core panel for phenotyping (Table S5).

#### Phenotyping of subset panel

The established subset was phenotyped for grain yield and grain quality‐related traits. Significant variations were observed across all the target traits. It was found that plant height varied from 58 to 160 cm, tiller number (4–19), days to flowering (74–105), panicle length (12–31.33 cm), primary branches per panicle (5–13), single plant yield (3.18–62.17 g), grain size (*l*/*b* ratio: 1.81–3.30), amylose content (17–33%), Fe concentration (9–19.8 ppm) and finally Zn concentration ranging from 11.50 to 26.30 ppm (Table S6).

**Figure 3:**
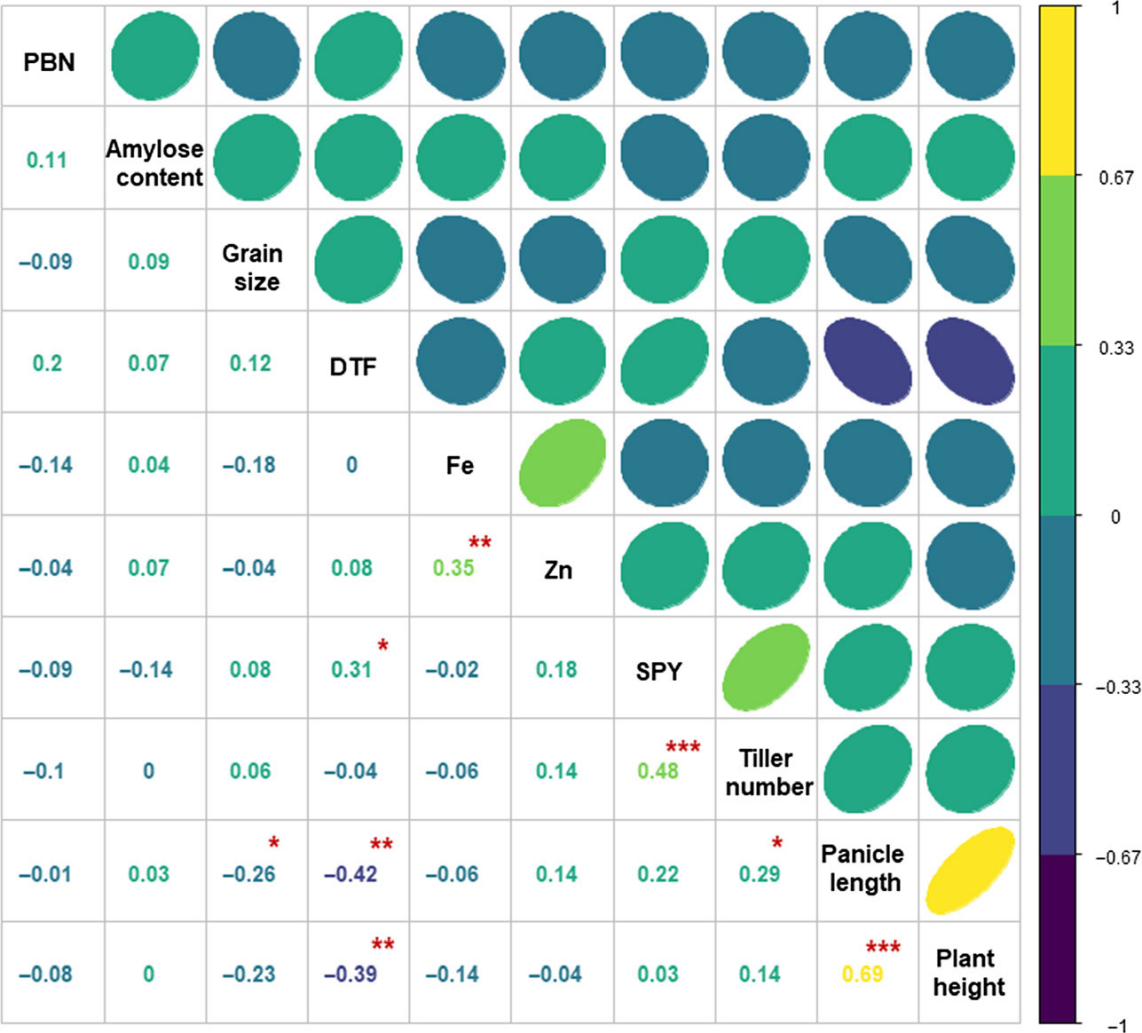
Correlation analysis of various grain yield and quality related traits in the subset of 3K RG panel. Days to flowering, tiller number and panicle length were positively associated with single plant yield. Plant height is negatively related with days to flowering and on the other hand positively linked with panicle length. It was observed that the panicle length and tiller number were positively correlated and was negatively related to days to flowering and grain size. Grain Fe and Zn concentration were positively correlated. DTF, Days to Flowering; PBN‐Primary branch number per panicle and SPY‐Single plant yield; *P < 0.05, **P < 0.01, ***P < 0.005.

Besides, correlation analysis was carried out to uncover the relationships among various traits (Figure 3). Panicle length was positively correlated with plant height and tiller number. However, negative correlation was found between panicle length and days to flowering and grain size. Highly significant positive correlation was observed between tiller number and plant yield. Similarly, positive relationship was observed between grain Fe and Zn concentration. No significant correlation was observed among grain quality and single plant yield.

#### Identification of superior haplotypes

**Figure 4:**
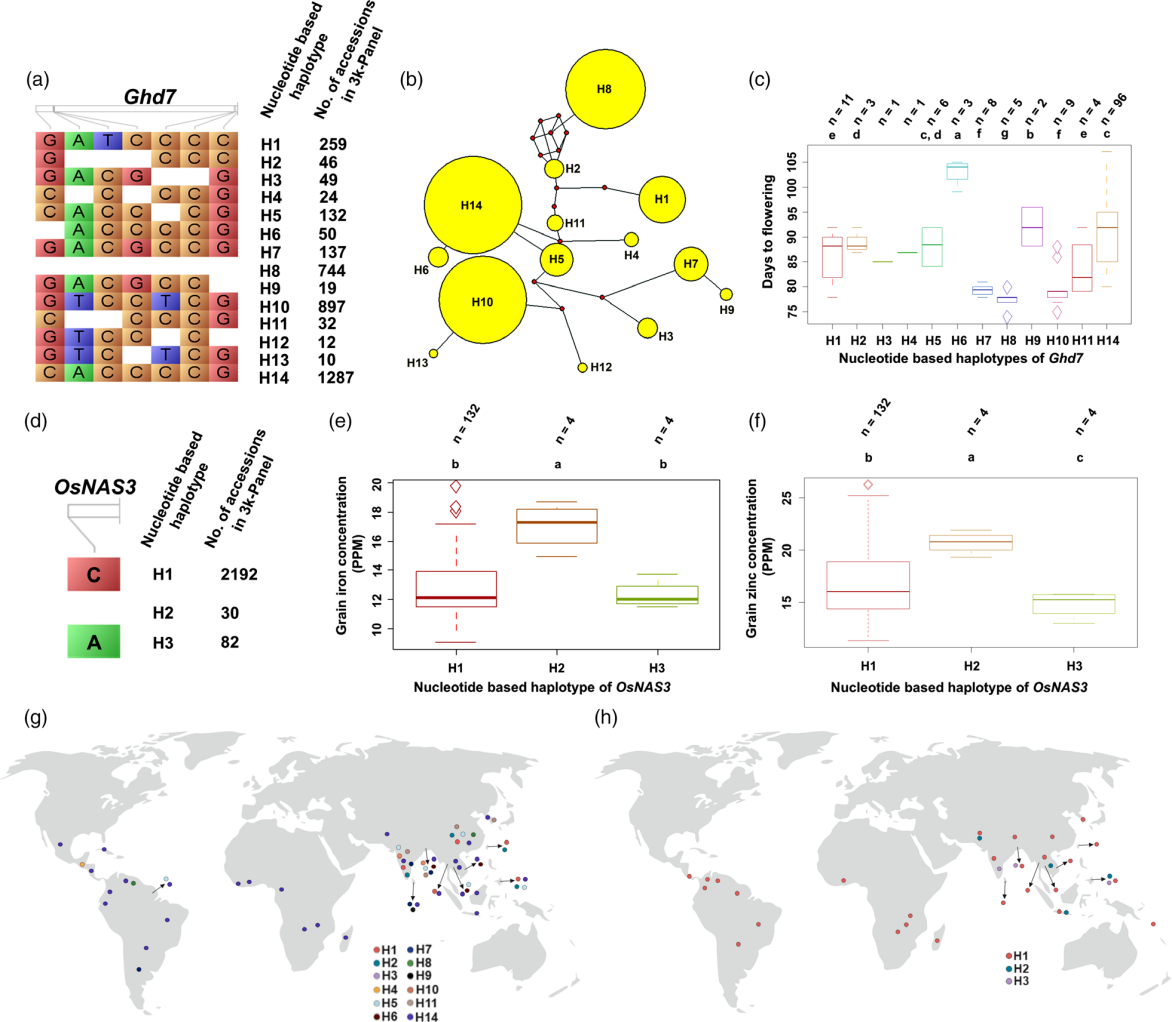
Haplotype analysis of *Ghd7* and *OsNAS3* across the 3K RG panel. (a) *Ghd7*, a key gene associated with heading date has about 14 haplotypes in the 3K RG panel with wide phenotypic variations. (b) *Ghd7*‐H8 was the most diverse one based on SNP and (c) interestingly was the earliest to flower, while *Ghd7*‐H6 took greater than 100 days to flower. (d) *OsNAS3* that influences grain Fe and Zn concentration has three haplotypes in the 3K RG panel with significant phenotypic variations in the subset. *OsNAS3*‐H2 had the highest grain (e) Fe and (f) Zn profile. The geographical distribution of various haplotypes of (g) *Ghd7* and (h) *OsNAS3*.

The phenotypic performance of various haplotypes for selected representative genes controlling yield and yield‐related traits in the subset of 3K RG panel consisting of 150 diverse lines was evaluated in two crop seasons. *Ghd7*, a key gene associated with heading date had 14 haplotypes in the 3K panel (Figure 4a). A total of 1287 accessions of the 3K RG panel possess *Ghd7*‐H14, while only 10 had *Ghd7*‐H13. Also, network analysis established sequence level variations and relationships among the identified haplotypes of *Ghd7* (Figure 4b). It was found that *Ghd7*‐H8 was the most diverse in comparison with the rest of them. Out of identified 14 haplotypes, 12 were only present in the subset panel and phenotyping of the subset revealed significant variations in days to flowering among the screened 12 haplotypes and it was categorized into eight significant groups based on Duncan's test (Figure 4c). Accessions with *Ghd7*‐H8 flowered as early as ~76 days, while the ones with *Ghd7*‐H6 flowered only after ~103 days. Interestingly, among the phenotyped 150 lines, the ones with *Ghd7*‐H8 were confined to China and Venezuela (Figure 4g). In addition, lines with *SNB*‐H9 and *TRX1*‐H9 was late flowering (94–103 days), *OsVIL3*‐H14 was medium duration type as they flowered in about 81–85 days, while *TOB1*‐H10 was flowered in just ~76 days (Table S6). Based on flowering time, *TOB1* and *OsTRX1* had two statistically significant haplotypes, while *SNB* and *OsVIL3* possessed five and four significant groups, respectively.

Furthermore, similar analysis was carried out for other target traits, such as, plant height, tiller number, panicle branching, grain yield, amylose content etc. About six haplotypes of *SD1* showed wide range of differences in plant height (Figure S5a). The germplasm lines with *SD1*‐H8 were about 90–110 cm tall, whereas the ones with *SD1*‐H1 were 130–150 cm in height. A total of five statistically significant groups were present among the subset panel for *SD1* haplotypes. Likewise, *MOC1*‐H9 and *MOC1*‐H10 had the highest (~10 tillers) and lowest number (~5 tillers) of tillers, respectively in the panel, which got categorized into three meaningful groups upon Duncan's analysis (Figure S5b). In regard to another tiller number associated gene, *IPA1*‐H14 (10–15 tillers) was found to be the superior most among the three statistically diverse haplotypes (Table S6). Based on *DEP1* and *DEP3* was with three diverse haplotypes, whereas *SP1* had just two significant groups. Also, *DEP3*‐H2, *DEP1*‐H2 and *SP1*‐H3 had the longest panicles (Figure S5c, Table S6). *LAX1*‐H5, *LP*‐H13 and *OSH1*‐H4 contained maximum branching (10–12 primary branches) in panicles (Figure S5d, Table S6). *LAX1* and *OSH1* had three significantly diverse haplotypes, while in the case of *LP* it was four. Importantly, *PHD1*‐H14, *AGO7*‐H15 and *ROC5*‐H2 contributed to the maximum yield (Figure S5e, Table S6). *PHD1* and *ROC5* also had three phenotypically diverse haplotypes as found by statistical analysis and on the other hand, *AGO7* possessed six diverse groups.

*OsNAS3*, one of the key genes influencing grain iron and zinc concentration possessed only three haplotypes across the 3K RG panel (Figure 4d) and all the haplotypes were present in the established sub‐set. About 2192 entries of the 3K RG panel had *OsNAS3*‐H1, whereas only 30 of them had *OsNAS3*‐H2. Interestingly, *OsNAS3*‐H2 had the highest Fe and Zn concentration in comparison with the other two haplotypes (Figure 4e,f). In the case of grain iron concentration, a total of two significant groups were present, while all the three‐haplotype formed separate groups for grain Zn concentration based on the statistical test. In the subset, the lines with *OsNAS3*‐H2 were from Cambodia, Indonesia, Philippines and Pakistan (Figure 4h). For grain quality, the accessions with *GS5*‐H4 were predominantly slender, whereas *GS5*‐H5 was found to be medium slender and *GS5*‐H9 were bold (Figure S5f). Expectedly, these three haplotypes of *GS5* belonged to three distinct categories based on a significance test. In addition, lines with *GW2*‐H2 were mostly medium slender. Similarly, *RSR1* was with four diverse haplotype groups which accounted for significant variations in grain amylose content across the screened subset. *RSR1*‐H2 was with low amylose content, while intermediate amylose content was found in *RSR1*‐H8 and high amylose content in *RSR1*‐H9, *RSR1*‐H10 haplotypes (Figure S5g).

### Haplotype analysis of target genes in mega‐varieties reveals the genetic basis of their superiority

Finally, to understand the superiority of the high yielding mega‐varieties, such as IR64, Swarna and IRRI 146, the haplotype combinations of the target genes were identified and analysed (Table 1). Interestingly, for five of the major traits, the same haplotypes were present across all the three high yielding varieties. For instance, all the three varieties possessed the same haplotypes for panicle length (*DEP3*‐H1 & *SP1*‐H2), primary branches per panicle (*LAX1*‐H8 & *LP*‐H8), grain size (*GS5*‐H5 & *GW2*‐H4), grain Fe and Zn concentration (*OsNAS3*‐H1). Also, among the three varieties, two (IR64 and IRRI 146) of them had *SD1*‐H9, while Swarna had *SD1*‐H5 for plant height. Similarly, for tiller number, *MOC1*‐H12 was present in Swarna, while the rest of them had *MOC1*‐H4. *IPA1*‐H12, *IPA1*‐H14, *IPA1*‐H10 was present in IR64, Swarna, and IRRI146, respectively. Also, for grain amylose content, *RSR1*‐H10 was in Swarna, whereas the others had *RSR1*‐H9. For grain yield, IR64 had *PHD1*‐H6 and *PHD1*‐H2 was found in Swarna and IRRI 146. Interestingly, all the three varieties had *OsAGO7*‐H12 and *ROC5*‐H5. In regard with days to flowering, IR64 and Swarna were found to have *Ghd7*‐H14, whereas *Ghd7*‐H5 was present in IRRI 146. All the three had *TOB1*‐H3 and *OsTRX1*‐H12. IR64 and IRRI146 carried *OsVIL3*‐H5, while Swarna had *OsVIL3*‐H14. Moreover, similar kind of analysis was carried out in an additional 16 selected accessions of the 3K RG panel and we report haplotype details in a total of 20 accessions (Table S7). Besides, efforts were made to decipher the haplotypes of the selected genes that were cloned in the earlier reports (Table S8). Interestingly, it was found that *Ghd7*‐H8, *SD1*‐H8 and *GS5*‐H5 were the originally cloned haplotype versions of these genes associated with heading date, plant height and grain size, respectively.

**Table 1:** Haplotype analysis of target genes in selected high yielding mega‐varieties and landraces

Traits	Target genes	Phenotype ranges of various haplotypes															Haplotypes in mega‐varieties and landraces					
		H1	H2	H3	H4	H5	H6	H7	H8	H9	H10	H11	H12	H13	H14	H15	IR64*	Swarna*	IRRI146*	Nona Bokra†	Dular†	N22†
Plant height (cm)	*SD1*	130–150	90–110	NV	80–115	115–155	NV	NV	90–110	60–165	NV	NV	NV	–	–	–	H9	H5	H9	H8	H9	H5
Tiller number	*MOC1*	5–8	NV	NV	5–13	11	NV	NV	NV	7–15	4–7	NV	4–18	NV	NV	–	H4	H12	H4	H9	H4	H7
	*IPA1*	7–12	NV	10	5	6	NV	NV	8	NV	7	NV	4–12	NV	10–15	NV	H12	H14	H10	H12	H14	H1
Days to flowering	*Ghd7*	77–93	87–93	85	87	84–93	98–104	78–81	74–80	88–95	75–89	79–93	NV	NV	80–106	–	H14	H14	H5	H10	H10	H7
	*SNB*	NV	79–107	NV	74–106	80	85–92	80–88	75–79	94–103	79	–	–	–	–	–	H2	H7	H4	H4	H8	H8
	*TOB1*	NV	NV	74–107	NV	NV	NV	NV	NV	NV	75–79	NV	–	–	–	–	H3	H3	H3	H3	H3	H3
	*OsVIL3*	88–92	NV	79–100	88	74–105	101	NV	NV	NV	75–107	NV	NV	80–106	81–85	NV	H5	H14	H5	H10	H10	H10
	*OsTRX1*	NV	92	NV	NV	NV	NV	NV	NV	94–99	NV	NV	74–107	NV	86	NV	H12	H12	H12	H12	H12	H12
Panicle length (cm)	*DEP3*	11–31	25–31	23–25	–	–	–	–	–	–	–	–	–	–	–	–	H1	H1	H1	H1	H2	H1
	*DEP1*	14–28	23–31	NV	NV	NV	19	NV	22	NV	NV	NV	NV	NV	11–27	NV	H1	H14	H14	H14	H1	H1
	*SP1*	15–31	11–26	23–27	–	–	–	–	–	–	–	–	–	–	–	–	H2	H2	H2	H1	H2	H2
Primary branches in panicle	*LAX1*	10	NV	10	NV	9–12	NV	NV	4–13	NV	4–7	–	–	–	–	–	H8	H8	H8	H10	H8	H8
	*OSH1*	5–10	NV	10	8–12	6	4–12	NV	NV	8	5–13	5–8	6	NV	NV	NV	H6	H6	H1	H6	H1	H6
	*LP*	NV	NV	NV	NV	4–11	5–9	NV	5–12	7	8–11	5–12	NV	8–13	10	NV	H8	H8	H8	H8	H11	H5
Single plant yield (g)	*PHD1*	19	3–38	NV	8	NV	3–18	NV	NV	NV	15	NV	NV	NV	17–62	NV	H6	H2	H2	H2	H2	H2
	*OsAGO7*	2–27	NV	2–14	NV	7–17	9–13	NV	3–10	20	NV	7	2–62	6	NV	16–33	H12	H12	H12	H12	H8	H1
	*ROC5*	4–14	16–30	NV	NV	2–62	NV	NV	NV	29	NV	NV	NV	NV	NV	–	H5	H5	H5	H5	H5	H5
Grain size	*GS5*	NV	NV	NV	2.4–4.1	1.8–3.2	NV	NV	NV	1.8–2.1	NV	NV	–	–	–	–	H5	H5	H5	H4	H4	H4
	*GW2*	NV	2.1–2.7	NV	1.8–4.1	–	–	–	–	–	–	–	–	–	–	–	H4	H4	H4	H4	H4	H4
Grain amylose content (%)	*RSR1*	11–33	11	NV	23–27	NV	24	23–28	10–22	20–31	23–28	NV	–	–	–	–	H9	H10	H9	H1	H7	H7
Grain Fe concentration (ppm)	*OsNAS3*	8–20	15–19	11–13	–	–	–	–	–	–	–	–	–	–	–	–	H1	H1	H1	H1	H1	H1
Grain Zn concentration (ppm)	*OsNAS3*	11–26	19–23	13–16	–	–	–	–	–	–	–	–	–	–	–	–	H1	H1	H1	H1	H1	H1

NV, not validated; –, Absent in 3K RG panel.

* Improved/mega‐varieties.

† Landraces/old varieties.

## Discussion

The rice 3K RG panel is a gold mine for harnessing haplotype diversity. In this study, more than 75% of the screened genes had rich diversity in the 3K RG panel thus confirming opportunities for improvement of major traits such as grain yield and grain quality, the two most important trait in rice, by *haplotype‐based breeding*. Also, about 10 of the most influencing traits contributing to grain yield and quality were chosen as representatives for validating the performance of various haplotypes of previously characterized key genes. For instance, *SD1* was proved to control plant height in rice (Monna *et al*., 2002), *MOC1* controls tillering (Li *et al*., 2003), *Ghd7* regulates heading date (Xue *et al*., 2008), *DEP3* determines panicle length (Qiao *et al*., 2011), *LAX1* directs panicle branching (Komatsu *et al*., 2003), *PHD1* influences grain yield (Li *et al*., 2011a), *GS5* plays a key role in determining seed size (Li *et al*., 2011b), *RSR1* regulates grain amylose content (Fu and Xue, 2010), while *OsNAS3* influences grain Fe and Zn concentration (Lee *et al*., 2009) etc.

We implemented an integrated strategy involving (i) meta‐analysis of target genes to facilitate the understanding of global expression profile of the chosen genes and (ii) phenotypic validation of various haplotypes to determine their superior combination. First, the analysis of spatiotemporal and developmental stage‐specific expression profile shed light into the expression pattern and co‐expression network of the target genes. Analysing the co‐expression network enhances the understanding of molecular framework regulating traits of interest and this would pave way for *systems biology driven breeding* in the coming years (Lavarenne *et al*., 2018). Recently, meta‐expression analysis coupled with co‐expression network predicted plausible role of about 2020 potential unannotated genes in rice (Chandran *et al*., 2018). Also, such analysis aids in understanding the roles of various genes and interactions among them at a systems level. We performed meta‐analysis of the publically available transcriptome datasets and found that *Ghd7* expressed the most in flag leaves and during booting stage, which very well corresponds with its previously characterized role for determining heading date.

Interestingly, *OsWAK120* (LOC_Os11g35860) was found to positively co‐express with *Ghd7*. In a previous study, *OsWAK* was present within genomic regions controlling number of panicles per plant and grain yield (Zhang *et al*., 2017). This might be one of the factors at molecular level, explaining the complex relationship between heading date and grain yield, which is to be validated in coming years. Another target gene, *OsNAS3* governing grain Fe & Zn concentration, predominantly expressed in panicle branches, at milk stage of grain filling, which explains its role. Moreover, *LTPL144* (LOC_Os10g40510), a protease inhibitor/seed storage/LTP family protein precursor, positively co‐expressed with *OsNAS3*. In a recent report, *LTPL144* was found within *qPCG10*/*qDC10* that plays a role in grain chalkiness and grain shape in rice (Mei *et al*., 2018). This suggests a plausible relationship among grain Fe & Zn concentration, grain shape and grain chalkiness. However, further confirmatory studies are to be carried out to validate and understand the co‐expression network. Deeper insights into the complex relationship among various target traits and the corresponding genes at the systems level would greatly aid in the selection of appropriate haplotypes for breeding and also facilitate the designing of *haplotype‐based breeding* scheme for tailored rice development in the coming years.

Second, phenotypic validation was carried out in the subset of the 3K RG panel to identify superior haplotypes for all of the 10 target traits. For instance, *SD1*‐H8 attributed to semi‐dwarf nature, *MOC1*‐H9 & *IPA1*‐H14 for high tiller number, *Ghd7*‐H14, *OsVIL3*‐H14 & *TRX1*‐H9 resulted in medium duration flowering, *DEP3*‐H2, *DEP1*‐H2 & *SP1*‐H3 for longer panicles, *LAX1*‐H5, *OSH1*‐H4 & *LAX1*‐H5 was with more number of panicle branches, *PHD1*‐H14, *AGO7*‐H15 & *ROC5*‐H2 had the highest single plant yield, *GS5*‐H4 for slender grains, intermediate amylose content was observed in *RSR1*‐H8 and finally *OsNAS3*‐H2 had high grain Fe & Zn profile. Furthermore, we also analysed the haplotype combinations in three of the high yielding mega varieties to understand the basis of their superiority. Interestingly, for three of the major traits *viz.,* panicle length, primary branch number per panicle and grain size they shared common haplotypes, which could be one of the major reasons for their superiority in terms of grain yield. In addition, it was also noticed that haplotypes for grain yield, tiller number etc., were among the good performing ones as validated across the panel. This approach opens an avenue for the identification of haplotypes that are responsible for the superior performance of the various mega varieties. Moreover, it was re‐confirmed that to result in high yield, it is very much necessary to have superior combinations of haplotypes associated with all of the component traits such as plant height, flowering date etc. For instance, even though ‘Dular’, ‘Nona Bokra’ and ‘N22’ were found to have *PHD1*‐H2 (haplotype for grain yield like Swarna), the presence of *Ghd7*‐H10 in Dular and Nona Bokra, *Ghd7*‐H7 in N22 makes them early flowering, hence possibly having a negative impact on grain yield. In support to this, correlation analysis also displayed a significant positive correlation between days to flowering and grain yield. This scenario suggests clearly that the assembly of desired combinations of haplotypes governing all the component traits in any genetic background is imperative to achieve yield and nutritional advantage in the future. Furthermore, in future, we emphasize that there is a need for whole genome re‐sequencing (WGRS) of newly developed elite rice varieties/breeding lines to analyse their haplotype diversity among key genes to understand the selection of key genes through genomic selection based approaches. This information will be useful for promotion and replacement of mega‐varieties.

Besides, the haplotype of certain cloned genes reported in earlier studies were investigated. *Ghd7* was originally cloned from ‘Zhengshan 97’ which is an early flowering line. As expected, we found that it was *Ghd7*‐H8 and was one among the early flowering haplotypes reported in this study. In addition, we also found two other early flowering haplotypes namely, *Ghd7*‐H7 and *Ghd7*‐H10. These evidences demonstrate the significance of haplotype‐based approach in terms of identification of superior/desired versions (haplotypes) of target genes that could be utilized in achieving high yield and nutrition.

We report best combination of haplotypes influencing the target traits *viz., SD1*‐H8 (plant height), *MOC1*‐H9 & *IPA1*‐H14 (tiller number), *DEP3*‐H2, *DEP1*‐H2 & *SP1*‐H3 (panicle length), *LAX1*‐H5, *OSH1*‐H4 & *LP*‐H13 (primary branch number per panicle), *PHD1*‐H14, *AGO7*‐H15, *ROC5*‐H2 (single plant yield), *OsNAS3*‐H2 (Fe & Zn concentration). With regard to days to flowering the reported haplotypes could be chosen based on the requirements, *Ghd7*‐H8 & *TOB1*‐H10 (early flowering), *Ghd7*‐H14, *OsVIL3*‐H14 & *TRX1*‐H9 (medium duration flowering) and *Ghd7*‐H6 & *SNB*‐H9 (late flowering). Similarly, based on the region of choice the haplotype for grain amylose content can be selected, *RSR1*‐H2 for low amylose content (suiting South East Asia), *RSR1*‐H8 for intermediate amylose content (suiting India, Nepal) and *RSR1*‐H9 to have high amylose content (for Bangladesh). In the same way, the haplotypes for grain size could also be chosen on the basis of preference, *GS5*‐H4 (slender), *GS5*‐H5 & *GW2*‐H2 (medium slender) and *GS5*‐H9 (bold). It was found that none of the analysed mega‐varieties possessed this ideal combination of haplotypes. It is evident that for plant height *SD1*‐H2, H4, H8 haplotypes are better than H9, H5 presently present in the three mega varieties. For number of tillers, *MOC1*‐H9 haplotype is better than H4, H12 present in mega varieties. For panicle length, *DEP3*‐H2, H3 haplotypes are better than the presently present H1 haplotype in the mega varieties and for single plant yield, *PHD1*‐H14 haplotype is better than H6, H2 haplotypes present in mega varieties. In the case for other genes also *viz., AGO7*, *ROC5, DEP1, SP1, OSH1, LP* etc., none of the analysed mega varieties carried superior haplotypes. This indicates that through *haplotype‐based breeding* we can further increase the yield over that of IR64, Swarna and IRRI 146 along with enhanced grain Fe and Zn concentration.

**Figure 5:**
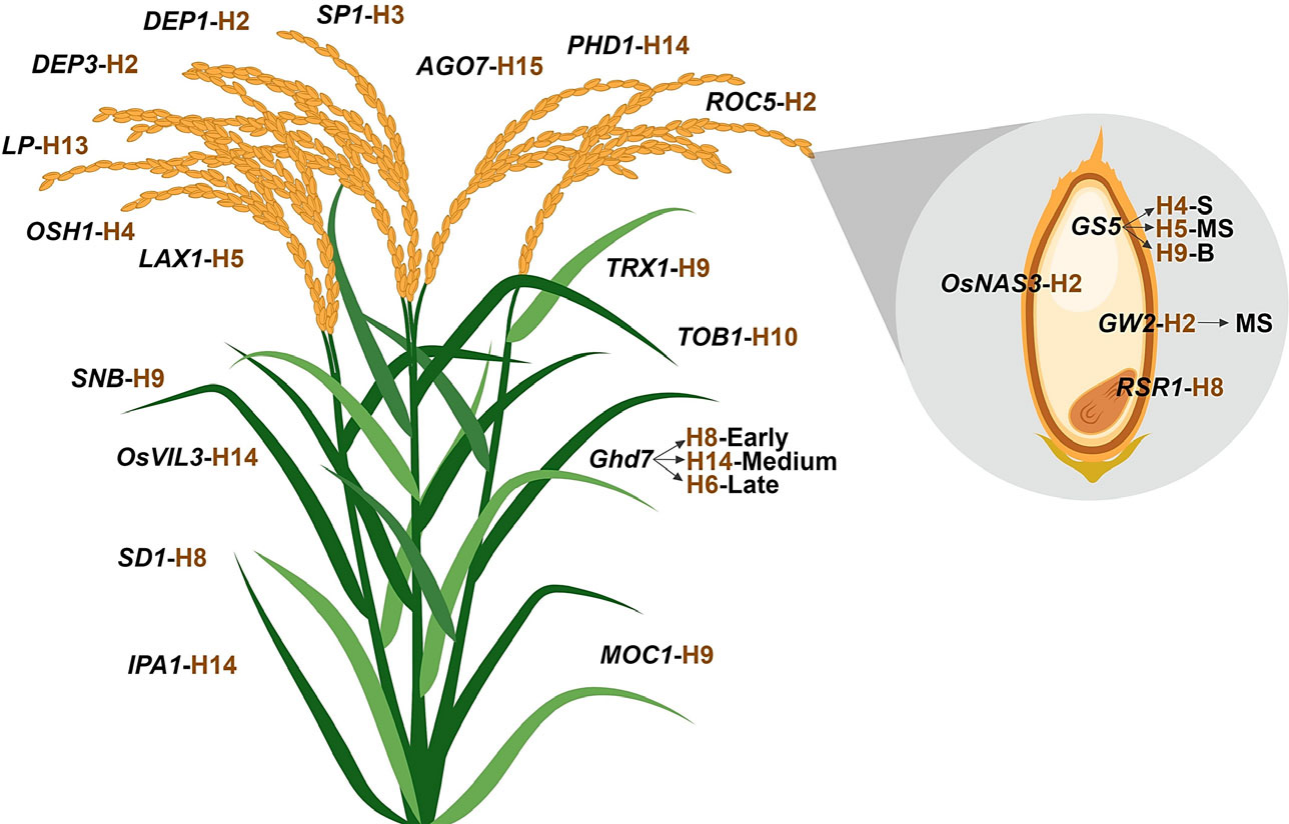
The tailored rice with superior haplotypes for grain yield and quality. The findings of this study could be employed to develop a designer rice genotype comprising superior haplotype combinations of the target genes such as *MOC1*‐H9 & *IPA1*‐H14 for higher tiller number, *Ghd7*‐H8 & *TOB1*‐H10 for early, *Ghd7*‐H14 & *OsVIL3*‐H14 for medium duration, *Ghd7*‐H6, *SNB*‐H9 & *TRX1*‐H9 for late flowering, *DEP3*‐H2, *DEP1*‐H2 & *SP1*‐H3 for long panicles, *SD1*‐H8 for semi‐dwarf nature, *LAX1*‐H5, *OSH1*‐H4 & *LP*‐H13 resulting in increased panicle branching, *PHD1*‐H14, *AGO7*‐H15 & *ROC5*‐H2 for high yield, along with *GS5*‐H4 for slender, *GS5*‐H5 & *GW2*‐H2 for medium slender, *GS5*‐H9 for bold grains, *RSR1*‐H8 for intermediate amylose content and *OsNAS3*‐H2 for increased Fe and Zn concentration in grains. B, Bold grain; MS, Medium slender type grain; S, Slender type grain.

In a nutshell, this study indicates the possibility of developing ‘tailored rice’ with increased yield that possesses desired eating quality, high Fe and Zn concentration, via *haplotype‐based breeding* (Figure 5). This study is the first step towards harnessing genetic diversity of previously cloned genes with validation across the subset of 3K RG panel.

However, in the future care should be taken to avoid unintended epistatic interactions when deciding the ideal haplotype combination. To address this, development of haplotype‐based near‐isogenic lines (Haplo‐NILs) in various genetic backgrounds is indispensable. This will be useful to determine any possible unintended effect on its performance regarding, (i) introgressed haplotypes and with genetic background markers loci (ii) Haplotype‐Haplotype interaction and (iii) interactions among background markers loci itself on different chromosomes influence the expression of complex traits.

In the coming years, *superior haplotype‐based genomic selection* (*Haplo‐GS*) powered by rapid generation advance/speed breeding that involves in the combining of all the superior haplotypes by using robust GS is expected to aid in the development of tailored rice suiting future food and nutritional demands. For the genotyping, superior haplotypes specific markers would be utilized to facilitate combining them into one background. The identification of accessions carrying multiple superior haplotypes associated with various traits would significantly fasten the process of tailored rice development. For instance, in this study we identified an accession, ‘IRIS_313‐10179’ carrying multiple superior haplotypes *viz., PHD1*‐H14, *AGO7*‐H15 for grain yield associated genes. Similarly, another accession ‘IRIS_313‐9936’ carrying both *MOC1‐*H9 & *IPA1*‐H14 genes associated with higher number of tillers per plant. In this manner, lines carrying multiple superior haplotypes can be identified and used in *Haplo‐GS* for rapidly combining several superior target haplotypes governing various major traits into one background for developing tailored rice suiting future food and nutritional demands. Besides, similar ideology could also be extended to other crops in coming years for harnessing the existing haplotype diversity.

## Conclusions

Haplotype analysis of 120 functionally characterized genes related to grain yield and quality clearly indicated the presence of extensive variations in 3K RG panel. The spatiotemporal and developmental stage specific transcriptome analysis using available datasets provided interesting insights such as the expression pattern of the target genes, positive co‐expression of *OsWAK120* with *Ghd7* and *LTPL144* with *OsNAS3*. Candidate gene based association analysis of 120 genes revealed that 21 previously cloned genes were strongly associated with the 10 major grain yield and quality traits. Importantly, upon phenotypic validation superior/desired haplotypes for each of the target trait *viz.,* plant height (*SD1*‐H8), productive tiller number (*MOC1*‐H9 & *IPA1*‐H14), flowering time (*Ghd7*‐H14, *OsVIL3*‐H14 & *TRX1*‐H9), panicle length (*DEP3*‐H2, *DEP1*‐H2 & *SP1*‐H3), primary branch number per panicles (*LAX1*‐H5, *LP*‐H13, *OSH1*‐H4), grain yield (*PHD1*‐H14, *AGO7*‐H15 & *ROC5*‐H2), grain size (*GS5*‐H4 & *GW2*‐H2), amylose content (*RSR1*‐H8) and grain Fe and Zn concentration (*OsNAS3*‐H2) were identified. Introgression of these superior haplotypes by the robust *haplotype‐based breeding* is a promising strategy for designing next generation rice varieties suiting future food and nutritional needs under shrinking cultivable land and rapidly increasing population along with fluctuating climatic conditions. We strongly believe that once realized, this strategy would pave way for future rice improvement.

## Materials and methods

### Haplotype analysis of functionally characterized genes across the 3K RG panel

For compiling the major cloned and characterized genes, two strategies were employed. First, the major genes influencing grain yield and quality were chosen from the ORGO database (Yamamoto *et al*., 2012). Furthermore, based on literature mining additional functionally characterized genes were selected. Following this, in‐built tool of SNP seek database (Mansueto *et al*., 2016) was utilized to conduct haplotype analysis for all the selected genes, by employing default parameters with Calinski criteria for k‐group determination. Nipponbare was used as the reference genome. All of the 3024 lines belonging to 12 sub‐populations namely, aro, aus, admix, ind1A, ind1B, ind2, ind3, indx, japx, subtrop, temp and trop was considered for the analysis. We utilized the ‘3kfiltered’ SNP set present in the SNP seek database for the entire analysis. The filtered was obtained from the Base SNP set by applying the following filtering criteria: (i) alternative allele frequency at least 0.01, (ii) proportion of missing calls per SNP at most 0.2 (Mansueto *et al*., 2016; http://snp-seek.irri.org/_download.zul) and this SNP set was already available in the SNP seek database which was directly utilized in this study. Haplotype analysis for all the genes have been carried out considering only the nonsynonymous SNPs that is the SNPs present in the exon region that results in amino‐acid change. Haplotypes of chosen representative genes were visualized in Flapjack (Milne *et al*., 2010). Finally, haplotype network analysis was conducted for a representative using Network 5 (Bandelt *et al*., 1999).

### Spatiotemporal and co‐expression analysis of the target genes

Genevestigator (Hruz *et al*., 2008) was utilized for the meta‐analysis of transcript expression profile for the short‐listed potential candidate genes. First, the spatiotemporal expression profile was uncovered, followed by the construction of co‐expression work for the target genes. Later, heat map for the spatiotemporal profile was generated using MeV tool. Similarly, the co‐expression network was visualized by Cytoscape 3.6.1 (Shannon *et al*., 2003).

### Phenotyping for yield and related traits in the 3K RG panel subset

Thirty‐day‐old seedlings of the selected germplasm lines from 3K RG panel were transplanted in the main field and planted at a spacing of 20 × 15 cm with a fertilizer dosage of 120–60–40 (N : P : K) kg/ha during wet season, 2017 and dry season, 2018 at ICRISAT experimental field, Hyderabad, India. The experimental plots were arranged in an alpha lattice design with six blocks and two replications maintained in each block. Standard agronomic practices were followed while growing the rice plants. Data were recorded from three randomly chosen plants in each plot for the following agronomic traits, *viz.,* days to 50% flowering (DFF), mean plant height (cm), number of productive tillers, grain yield per plant (g), panicle length (cm) and number of primary branches per panicle.

### Evaluation of grain quality related traits

#### Quantifying grain Zn and Fe concentration using Bruker S2 RANGER

The accessions were harvested after attaining maturity, threshed, dried up to 12% moisture content, cleaned for dirt and foreign materials. The cleaned seeds were dehusked with the help of rice huller (Model: H‐750 series, Krishi International, Hyderabad, India) to get brown rice (hulled rice). Again, the brown rice was cautiously cleaned for dust and other impurities and ultimately pure brown rice were used for the quantification of Fe and Zn concentration using EDXRF (X‐ray Fluorescent, Model: Bruker S2 ranger fitted with a 28 place auto sampler). Measurement conditions were followed as per the recommendation given by the manufacturer. Total analysis time for each sample was 186 s, which included 60 s acquisition times for the separate Zn and Fe conditions as well as 66 s ‘dead time’ during which the EDXRF established each measurement condition. Scans were conducted in sample cups having the diameter of 21 mm Al cups combined with polypropylene inner cups sealed at one end with 4 μm Poly‐4 XRF sample film. Cups containing samples were gently shaken to evenly distribute grains. Preliminary studies showed that, a sample depth of ≥6 mm was required for maximum recovery of Zn and Fe signal in rice. According to the manufacturer, the Bruker S2 Ranger scans a circle of 21 mm diameter with the sample spinner on. All scans in this study were performed in this mode, so the scanned area was 346 mm. Sampling volumes were therefore ~2.1 cm^3^ for Zn and Fe analysis.

#### Grain size

Kernel length and kernel breadth were measured using graph sheets and further evaluated based on Standard Evaluation System of Rice (IRRI, 2013) across the chosen panel.

#### Rapid screening of amylose content in grains (Cut Grain Dip method)

A single well‐matured rice grain was cut into two half halves horizontally in the middle with a pair of scissors. Then a drop of (KI‐I) solution in the ratio of 6 : 1 (standardized ratio; Agasimani *et al*., 2013) was placed on the cut half of the grain by using micropipette. At most care has been taken to retain the droplet on the cut end. After placing the droplet, the time taken to colour change in white to deep blue colour was noted with the help of electronic stopwatch. The observations were made for five cut grains and average time was estimated for each genotypes studied. Then, the amylose content of each genotype was predicted based on the available ready reckoner (Agasimani *et al*., 2013).

### Candidate gene based association analysis and identification of superior haplotypes

Candidate gene based association analysis was conducted using MLM model in GAPIT, taking both kinship (K) and population structure (Q) in to account, for identifying strongly associated previously cloned genes with the corresponding target traits. All the SNPs present within the candidate genes was considered for the association analysis and this information was obtained from the SNP‐seek database (http://snp-seek.irri.org/_download.zul). A threshold *P* value <0.01 was implemented significant SNP‐trait association. The resultant significantly associated candidate genes was further used for determining superior haplotypes. For this, the phenotyped subset was categorized on the basis of haplotypes for all the strongly associated genes separately. Then, the corresponding trait's mean (haplotype‐wise) was used to identify the superior haplotype of a particular associated gene.

### Statistical analysis

All the recorded phenotypic data were from two crop seasons. Duncan analysis was performed to test statistical significance to understand phenotypic performance of each haplotype. Different alphabets denote significant difference and vice‐versa. Furthermore, only the haplotypes validated in at least two lines were considered for statistical analysis.

## Conflict of interest

The author(s) declare that they have no competing interests.

## Supporting information


**Figure S1** Haplotype frequency analysis for selected target genes and haplotype distribution based on sub‐population. (a) *SD1*, (b) *MOC1*, (c) *Ghd7*, (d) *DEP3*, (e) *LAX1*, (f) *PHD1*, (g) *GS5*, (h) *RSR1*, (i) *OsNAS3*. The numbers in the pie chart denote haplotype frequency in %.
**Figure S2** Analysing the spatiotemporal expression profile of the target genes using publically available transcriptome datasets.
**Figure S3** Co‐expression network of (a, b) *Ghd7* and (c, d) *OsNAS3* sheds light on the nature of positively correlated genes.
**Figure S4** Co‐expression network of the target genes related with grain yield and quality. (a) *SD1*, (b) *DEP3*, (c) *LAX1*, (d) *PHD1*, (e) *GS5*, (f) *RSR1*.
**Figure S5** Identification of superior haplotypes for the target yield and grain quality related traits. Significant phenotype diversity was explained by the various haplotypes of the target genes in the subset of 3K RG panel. The mean data across two seasons are depicted for (a) plant height, (b) tiller number, (c) panicle length, (d) number of primary branches in panicles, (e) single plant yield, (f) grain size and (g) grain amylose content.
**Table S8** Haplotype analysis of previously cloned versions of target genes.

**Table S1** Major functionally characterized genes governing grain yield and quality related traits in rice along with the number of haplotypes in the 3K RG panel.

**Table S2** Candidate gene based association study provided insights into 21 strongly associated genes with the target grain yield and quality traits in rice.

**Table S3** Haplotype analysis of the key genes governing the target traits across the 3K RG panel.

**Table S4** Functional categorization of the co‐expressed genes.

**Table S5** Country wise list of 3K RG panel subset utilized for phenotyping the target traits for two seasons.

**Table S6** Haplotype‐phenotype relationship for the 21 strongly associated genes influencing the 10 target grain yield and quality traits.

**Table S7** Haplotype analysis of target genes in selected accessions of 3K RG panel.
