# Supplementary material for: Haplotype analysis of key genes governing grain yield and quality traits across 3K RG panel reveals scope for the development of tailor‐made rice with enhanced genetic gains
Source: Plant Biotechnol J. 2019 Feb 15;17(8):1612–22. doi: 10.1111/pbi.13087 (PMC6662101; doi:10.1111/pbi.13087)
Supplement: Supplementary file 1 — Figure S1 Haplotype frequency analysis for selected target genes and haplotype distribution based on sub‐population. (a) SD1, (b) MOC1, (c) Ghd7, (d) DEP3, (e) LAX1, (f) PHD1, (g) GS5, (h) RSR1, (i) OsNAS3. The numbers in the pie chart denote haplotype frequency in %. Figure S2 Analysing the spatiotemporal expression profile of the target genes using publically available transcriptome datasets. Figure S3 Co‐expression network of (a, b) Ghd7 and (c, d) OsNAS3 sheds light on the nature of positively correlated genes. Figure S4 Co‐expression network of the target genes related with grain yield and quality. (a) SD1, (b) DEP3, (c) LAX1, (d) PHD1, (e) GS5, (f) RSR1. Figure S5 Identification of superior haplotypes for the target yield and grain quality related traits. Significant phenotype diversity was explained by the various haplotypes of the target genes in the subset of 3K RG panel. The mean data across two seasons are depicted for (a) plant height, (b) tiller number, (c) panicle length, (d) number of primary branches in panicles, (e) single plant yield, (f) grain size and (g) grain amylose content. Table S8 Haplotype analysis of previously cloned versions of target genes. [file PBI-17-1612-s005.docx]

**SUPPORTING INFORMATION**

**Full title**

Haplotype analysis of key genes governing grain yield and quality traits across 3K RG panel reveals scope for the development of tailor-made rice with enhanced genetic gains

**Running title**

Haplotype-based breeding for tailored rice development

Ragavendran Abbai^1†^, Vikas Kumar Singh^1†^, Vishnu Varthini Nachimuthu^1^, Pallavi Sinha^2^, Ramchander Selvaraj^1^, Abhilash Kumar Vipparla^1^, Arun Kumar Singh^1^, Uma Maheshwar Singh^1^, Rajeev K Varshney^2^, Arvind Kumar^1, 3*^

^1^International Rice Research Institute, South-Asia Hub, ICRISAT Campus, Hyderabad-502324, India

^2^Centre of Excellence in Genomics and Systems Biology, International Crops Research Institute for the Semi-Arid Tropics, Hyderabad-502324, India

^3^International Rice Research Institute, DAPO BOX 7777, Metro Manila, Philippines

**Short Summary:** This study involves the haplotype analysis of 120 previously characterized genes governing grain yield and grain quality traits in rice utilizing 3K RG panel. We report superior haplotypes for each of the selected target traits upon phenotypic validation in the subset of 3K RG panel. This approach opens the avenue for developing ‘superior haplotype based tailored rice’ for achieving higher yield and genetic gains in rice.

^†^These authors contributed equally

^*^Author for Correspondence

Arvind Kumar

International Rice Research Institute, DAPO BOX 7777, Metro Manila, Philippines

Telephone: +63 2580 5600

Fax: +63 2 580 5699, +63 2 845 0606

Email: [a.kumar@irri.org](mailto:a.kumar@irri.org)


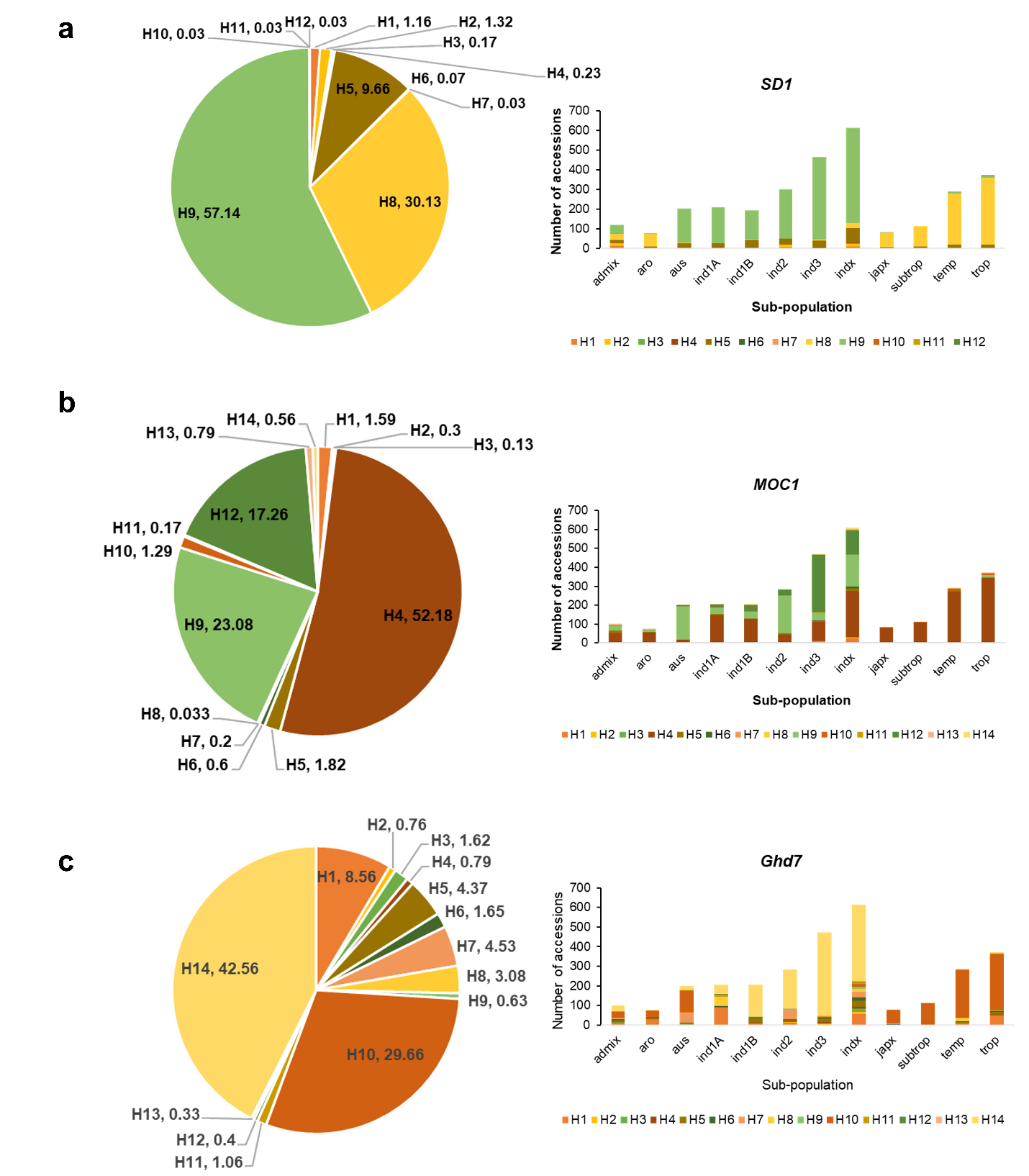


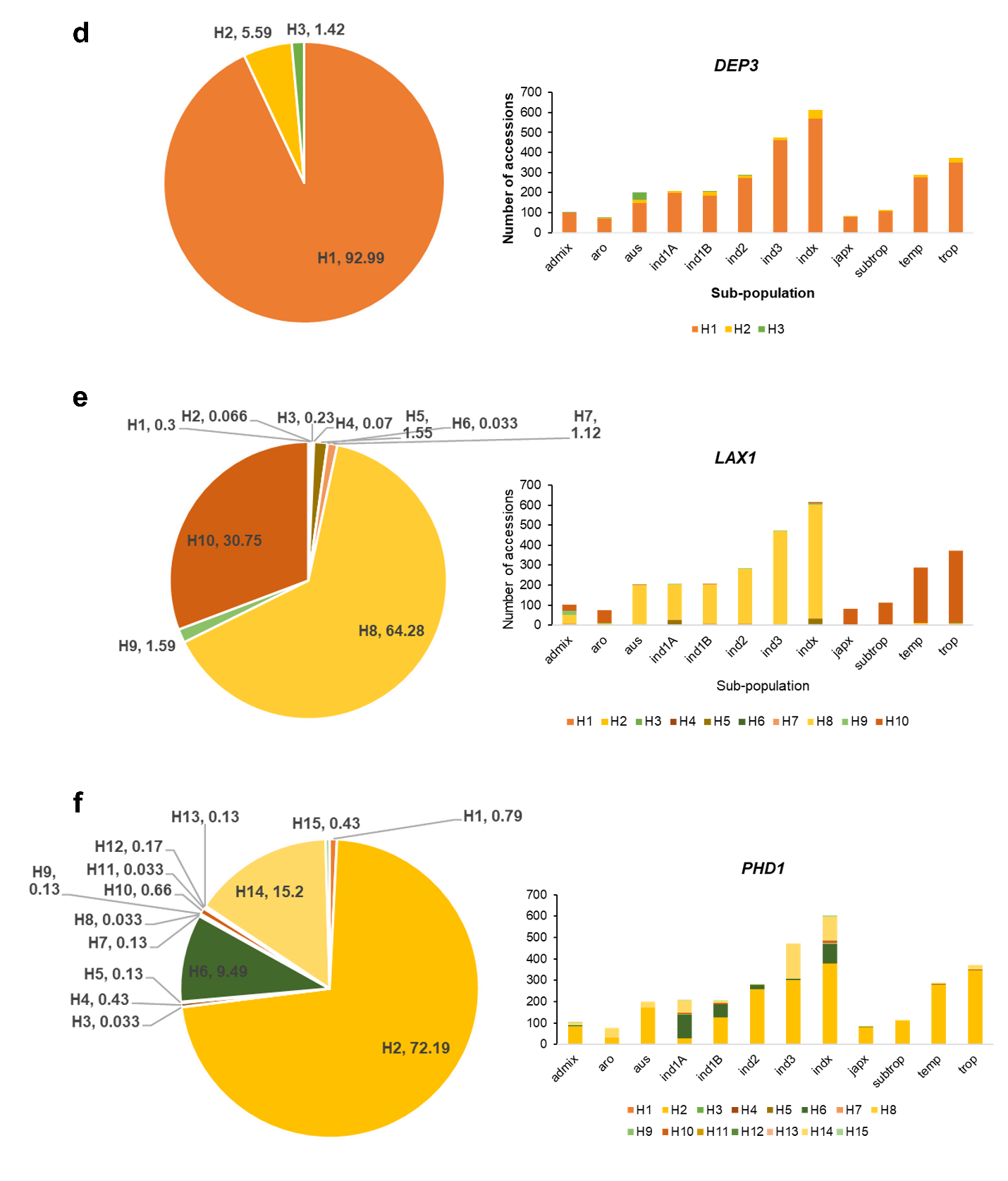


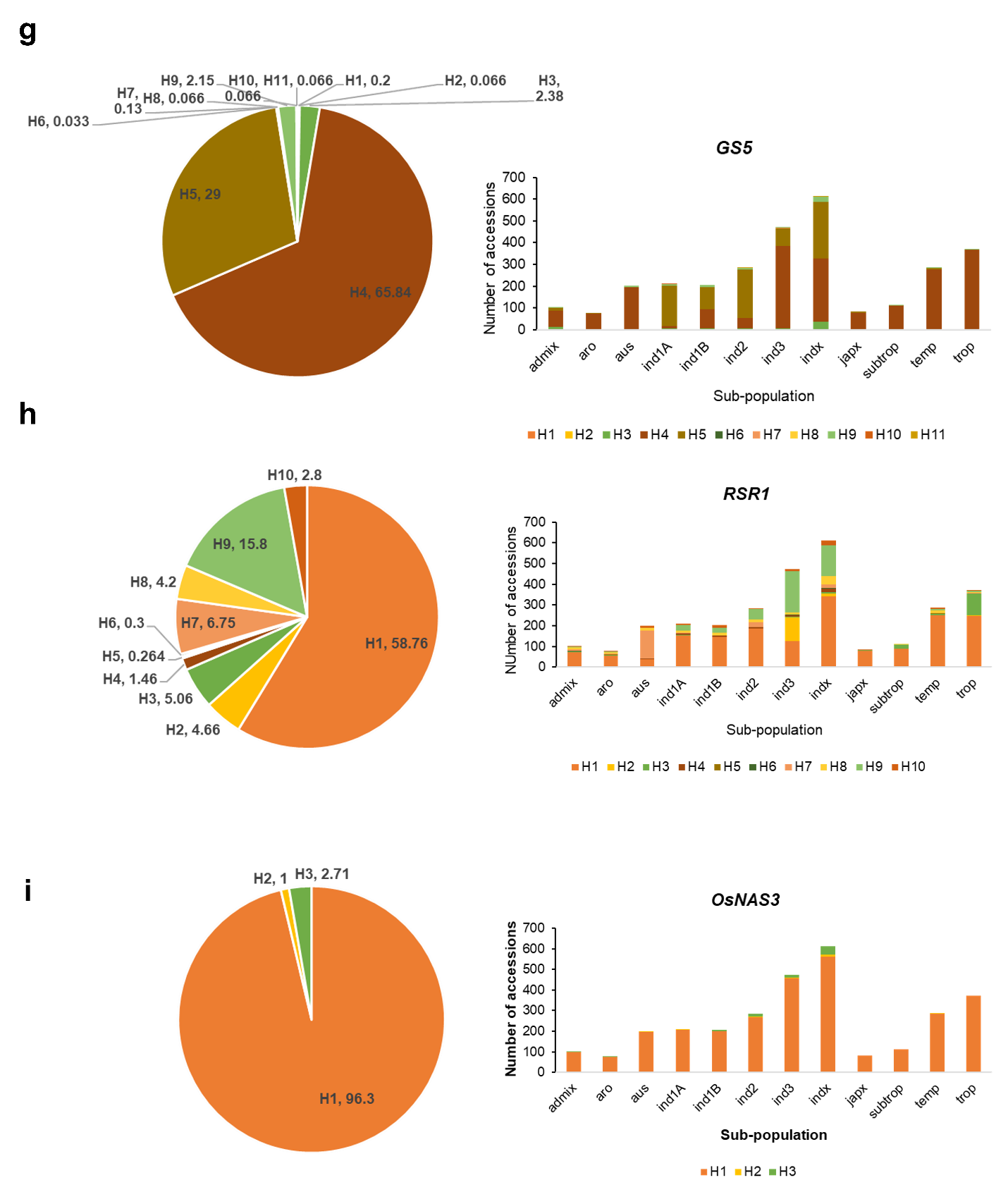


**Figure S1 Haplotype frequency analysis for selected target genes and haplotype distribution based on sub-population.** (a) *SD1*, (b) *MOC1*, (c) *Ghd7*, (d) *DEP3*, (e) *LAX1*, (f) *PHD1*, (g) *GS5*, (h) *RSR1*, (i) *OsNAS3*.

Note: The numbers in the pie chart denote haplotype frequency in %


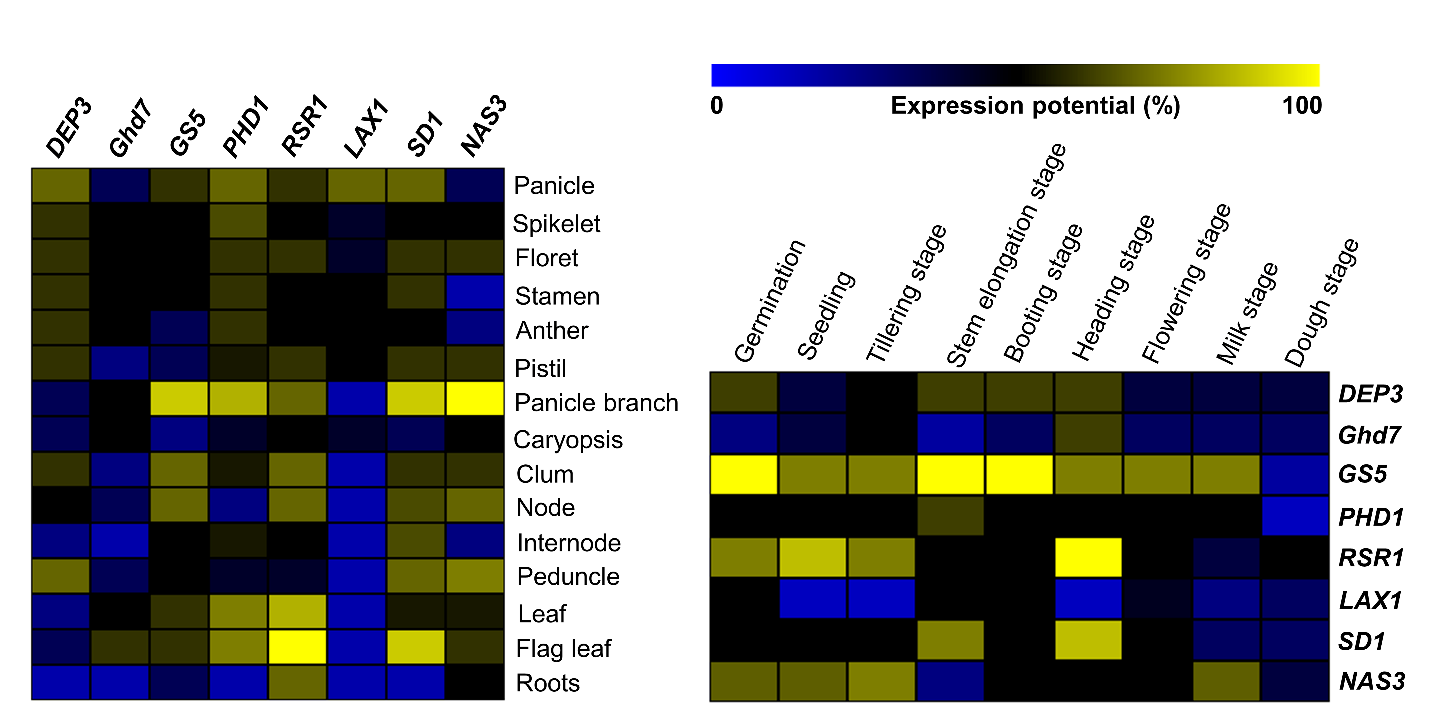


**Figure S2. Analyzing the spatiotemporal expression profile of the target genes using publically available transcriptome datasets.**


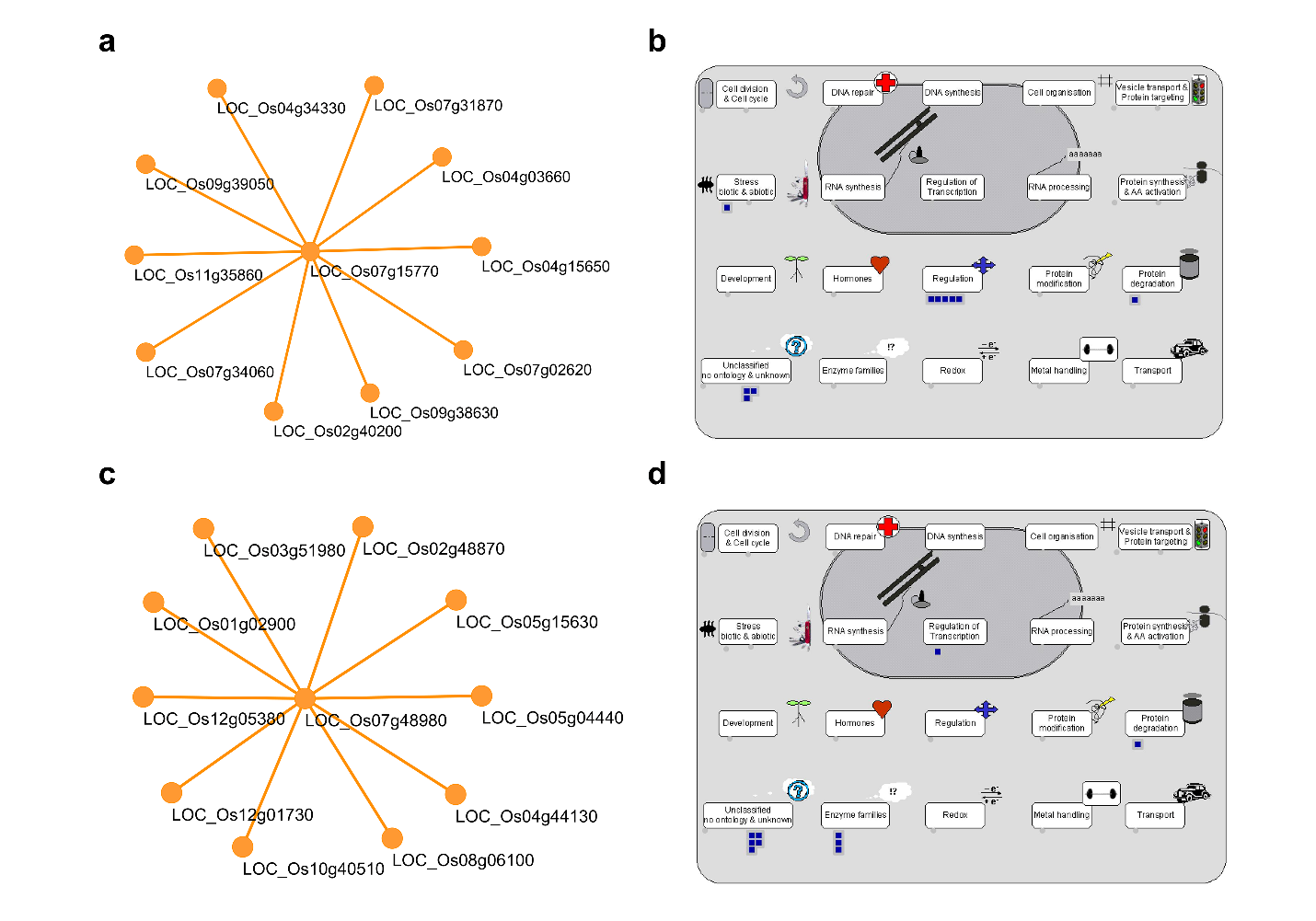


**Figure S3. Co-expression network of (a, b) *Ghd7* and (c, d) *OsNAS3* sheds light on the nature of positively correlated genes.**


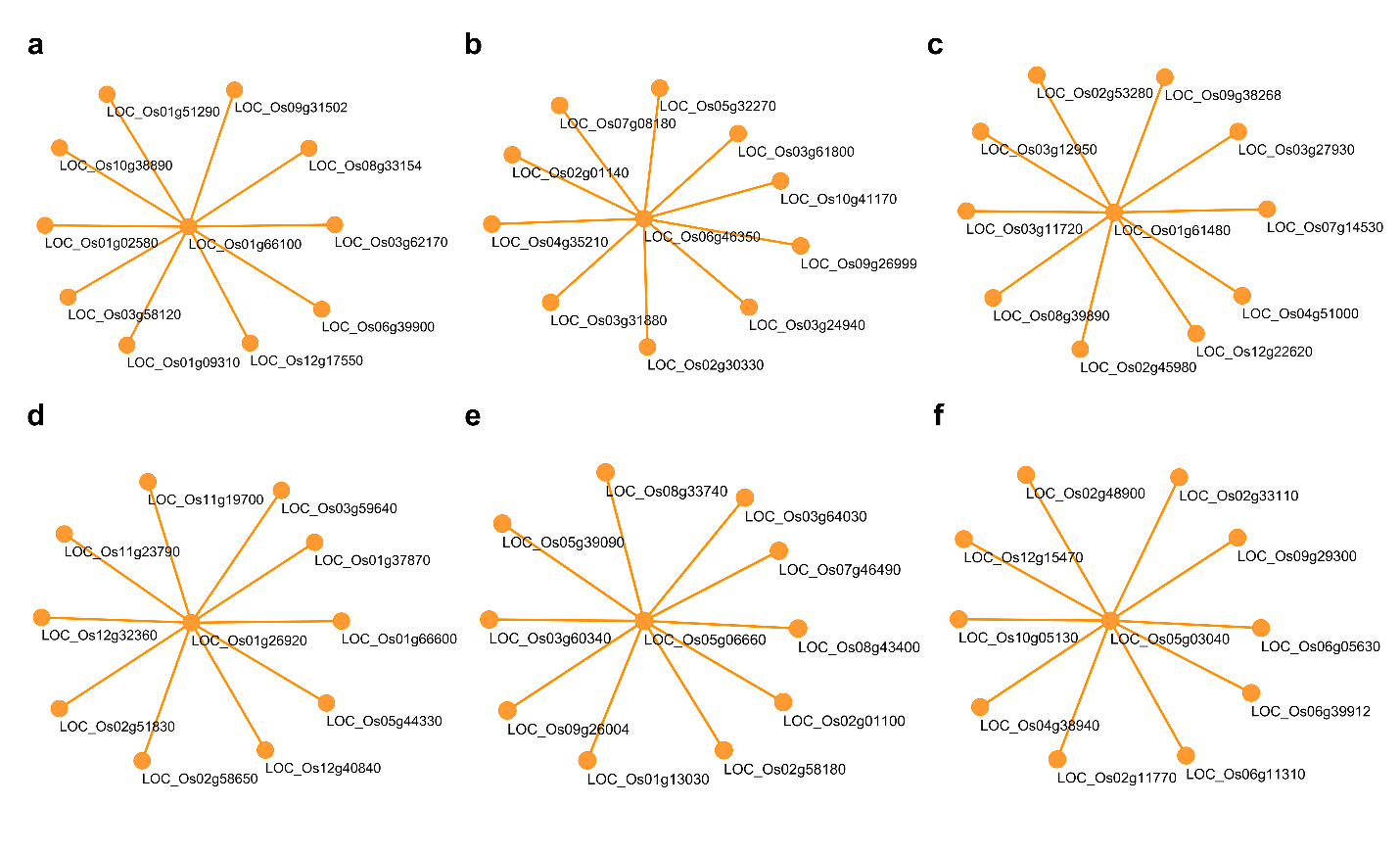


**Figure S4. Co-expression network of the target genes related with grain yield and quality.**

(a) *SD1*, (b) *DEP3*, (c) *LAX1*, (d) *PHD1*, (e) *GS5*, (f) *RSR1*


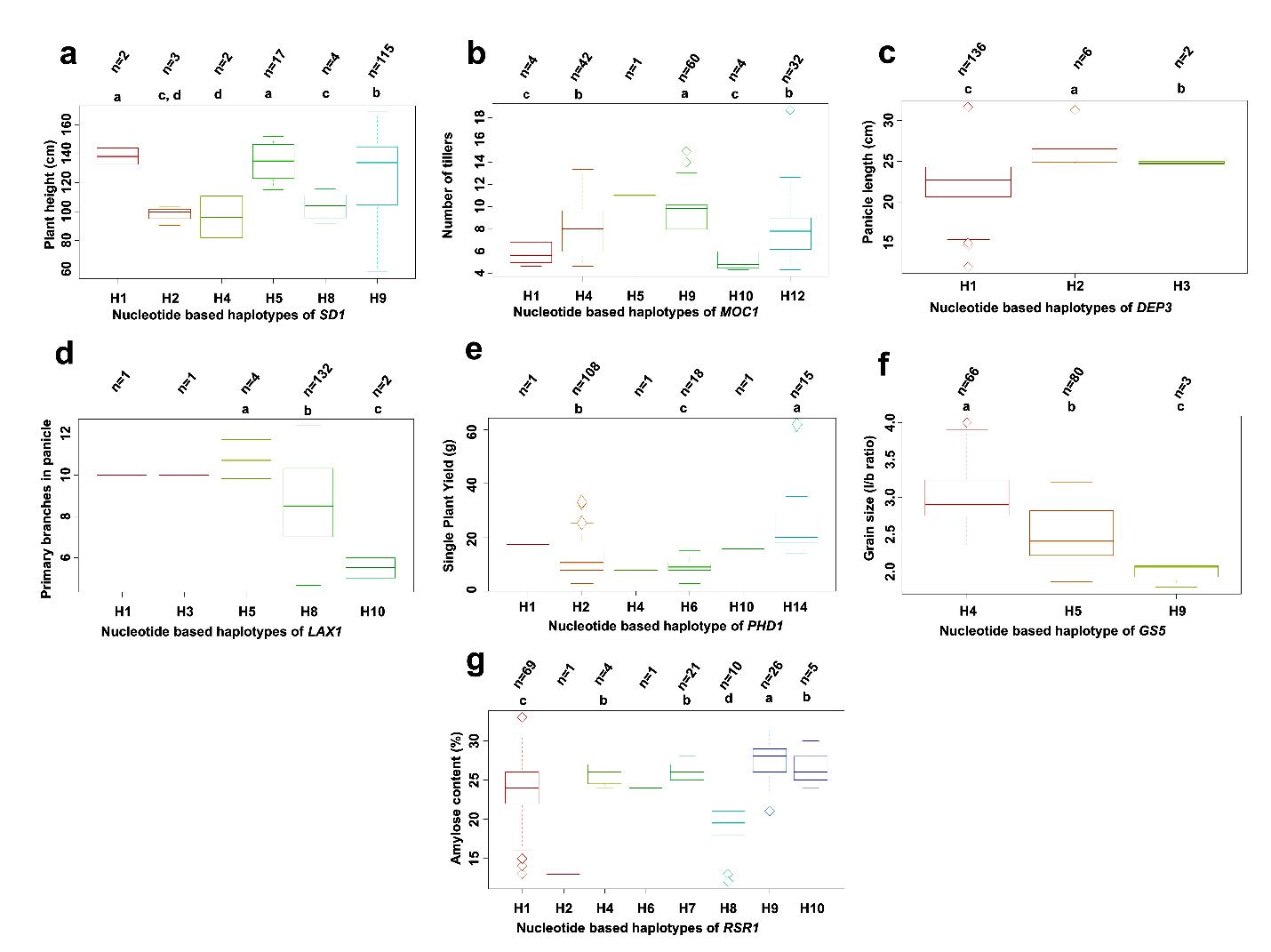


**Figure S5. Identification of superior haplotypes for the target yield and grain quality related traits.**

Significant phenotype diversity was explained by the various haplotypes of the target genes in the subset of 3K RG panel. The mean data across two seasons is depicted for (a) Plant height, (b) tiller number, (c) panicle length, (d) number of primary branches in panicles, (e) single plant yield, (f) grain size and (g) grain amylose content.

**Table S1** Major functionally characterized genes governing grain yield and quality related traits in rice along with the number of haplotypes in the 3K RG panel.

**(Supplied as datasheet)**

**Table S2** Candidate gene based association study provided insights into 21 strongly associated genes with the target grain yield and quality traits in rice.

**(Supplied as datasheet)**

**Table S3** Haplotype analysis of the key genes governing the target traits across the 3K RG panel.

**(Supplied as datasheet)**

**Table S4** Functional categorization of the co-expressed genes.

**(Supplied as datasheet)**

**Table S5** Country wise list of 3K RG panel subset utilized for phenotyping the target traits for two seasons.

**(Supplied as datasheet)**

**Table S6** Haplotype-phenotype relationship for the 21 strongly associated genes influencing the 10 target grain yield and quality traits.

**(Supplied as datasheet)**

**Table S7** Haplotype analysis of target genes in selected accessions of 3K RG panel

**(Supplied as datasheet)**

**Table S8** Haplotype analysis of previously cloned versions of target genes

| **Gene** | **Haplotype** | **Accession** | **Original report** |
| --- | --- | --- | --- |
| *SD1* | H8 | Milyang 23 | doi.org/10.1093/dnares/9.1.11 |
| *MOC1* | NA | H89025 | 10.1038/nature01518 |
| *IPA1* | NA | SNJ & Ri22 | 10.1038/ng.591 |
| *Ghd7* | H8 | Zhengshan 97 | 10.1038/ng.143 |
| *OsVIL3* | NA | T-DNA insertion mutant in A654 | 10.1093/mp/sss096 |
| *TRX1* | NA | T-DNA tagging in Donjin and Hwayoung | 10.1104/pp.113.228049 |
| *TOB1* | NA | EMS mutant (M2) of T65 | doi.org/10.1105/tpc.111.094797 |
| *SNB* | NA | T-DNA tagging in Donjin | 10.1111/j.1365-313X.2006.02941.x |
| *DEP3* | NA | Hwacheong | 10.1007/s00122-011-1543-6 |
| *DEP1* | NA | Shennong 265, Wuyunjing 7, Q169 and W101 | 10.1038/ng.352 |
| *SP1* | NA | Gamma radiation mutant of Zhonghua11 | 10.1111/j.1365-313X.2009.03799.x |
| *LAX1* | NA | Shinriki | 10.1073/pnas.1932414100 |
| *OSH1* | NA | MNU-mutagenized T65 population | doi.org/10.1105/tpc.111.090050 |
| *LP* | NA | 60Co γ‐ray‐irradiated Zhonghua 11 | 10.1111/j.1467-7652.2011.00610.x |
| *PHD1* | NA | Mutant in Nipponbare background | 10.1371/journal.pgen.1002196 |
| *AGO7* | NA | T-DNA insertion mutant in A654 | 10.1007/s00425-006-0472-0 |
| *ROC5* | NA | T-DNA insertion mutant | 10.1104/pp.111.176016 |
| *GS5* | H5 | Zhengshan 97 | 10.1038/ng.977 |
| *GW2* | NA | WY3 | 10.1038/ng2014 |
| *RSR1* | NA | Mutant in Zhonghua 11 background | 10.1104/pp.110.159517 |
| *OsNAS3* | NA | Mutant in Dongin background | 10.1073/pnas.0910950106 |

NA-Accession absent in 3K panel
